# Retraction Note: Production and bioprocessing of epothilone B from Aspergillus niger, an endophyte of Latania loddegesii, with a conceivable biosynthetic stability: anticancer, anti-wound healing activities and cell cycle analysis

**DOI:** 10.1186/s12934-026-03139-y

**Published:** 2026-10-07

**Authors:** Sara Refaat, Eman Fikry, Nora Tawfeek, Ashraf S. A. El-Sayed, Maher M. El-Domiaty, Azza M. El-Shafae

**Affiliations:** 1 Pharmacognosy Department, Faculty of Pharmacy, Zagazig University, Zagazig, 44519 Egypt; 2 Enzymology and Fungal Biotechnology Lab, Botany and Microbiology Department, Faculty of Science, Zagazig University, Zagazig, 44519 Egypt

**Retraction Note:** **Microbial Cell Factories (2024) 23:229**

https://doi.org/10.1186/s12934-024-02495-x

The Editor-in-Chief has retracted this article. After publication, concerns were raised regarding the data presented in the figures, specifically:

- Figs. 2E, 5 and 6 appear to share control group data with the authors’ other article [1];
- Fig. 5A EPT treated cells (HepG-2) 24 h image appears highly similar to Fig. 11A Doxorubicin (top image) in [2], stretched vertically;
- Fig. 5A EPT treated cells (HepG-2) 72 h image appears to overlap with Fig. 11A Purified EPT (top image) in [2].

The authors have stated that the control groups were shared between this article and [1] as the experiments were performed together, but have not been able to address the other concerns or provide the underlying raw data upon request. The Editors-in-Chief therefore no longer have confidence in the presented data.

Ashraf S.A. El-Sayed does not agree with this retraction. The publisher has not been able to obtain a current email address for Azza M. El-Shafae. The other authors have not responded to any correspondence from the editor or publisher about this retraction.
